# Incidence of Recurrent Respiratory Papillomatosis (RRP) in Denmark and Sweden During 2000-2023: Two Nationwide Cohort Studies in Children and Young Adults

**DOI:** 10.1093/ofid/ofag290

**Published:** 2026-06-16

**Authors:** Karin Sundström, Christian Munk, Jiangrong Wang, Kirsten Frederiksen, Sara Nordqvist Kleppe, Parag Mahale, Joseph E Tota, Susanne K Kjær

**Affiliations:** Center for Cervical Cancer Elimination, Dept of Clinical Science, Intervention and Technology, Karolinska Institutet, Stockholm, Sweden; Clinical Pathology and Cancer Diagnostics, Medical Diagnostics Karolinska, Karolinska University Hospital, Stockholm, Sweden; Unit of Virus, Lifestyle and Genes, Danish Cancer Institute, Copenhagen, Denmark; Center for Cervical Cancer Elimination, Dept of Clinical Science, Intervention and Technology, Karolinska Institutet, Stockholm, Sweden; Statistics and Data Analysis, Danish Cancer Institute, Copenhagen, Denmark; Center for Cervical Cancer Elimination, Dept of Clinical Science, Intervention and Technology, Karolinska Institutet, Stockholm, Sweden; Department of Epidemiology, Biostatistics and Research Decision Sciences, Merck Research Laboratories, Merck & Co., Inc., Rahway, New Jersey, USA; Department of Epidemiology, Biostatistics and Research Decision Sciences, Merck Research Laboratories, Merck & Co., Inc., Rahway, New Jersey, USA; Unit of Virus, Lifestyle and Genes, Danish Cancer Institute, Copenhagen, Denmark; Department of Gynecology, Copenhagen University Hospital, Rigshospitalet, Copenhagen, Denmark

**Keywords:** HPV, HPV vaccine, human papillomavirus, laryngeal papillomatosis, recurrent respiratory papillomatosis

## Abstract

**Introduction:**

Recurrent respiratory papillomatosis (RRP), primarily caused by human papillomavirus (HPV) types 6 or 11, is a rare but high-morbidity condition. Nationwide estimates of the incidence of RRP are scarce, and we aimed to estimate the incidence of RRP among children and young adults in Denmark (2000-2023) and Sweden (2000-2021).

**Methods:**

Using the Danish Pathology Register and the Swedish Patient Register, we identified all individuals with a first-time occurrence of RRP (definition in Denmark: histologically confirmed, topography code T24 plus SNOMED codes for papillomas; Sweden: International Classification of Diseases-10 code D14.1A for papillomas). Incidence rates were calculated as number of RRP cases per 100 000 person-years (py), stratified by country, sex, and age groups at onset (0-14/juvenile 15-29/adult). Trends in incidence were evaluated in the period before and after introduction of HPV vaccination using Poisson regression to estimate the annual percentage change (APC) and 95% confidence intervals (CI).

**Results:**

We found juvenile-onset RRP incidences of 0.14 to 0.20/100 000 py as well as adult-onset RRP incidences at 0.19 to 0.40/100 000 py for women and 0.52 to 0.68/100 000 py for men in the postvaccine introduction period. Danish data showed a decreasing trend in RRP during the period following implementation of HPV vaccination, especially among young adults (women: APC = −11.5 [95% CI, −20.9 to −0.9]; men: APC = −11.0 [95% CI, −16.6 to −4.9]), whereas Swedish data did not show any clear decrease in RRP over time.

**Conclusions:**

The incidence of RRP is decreasing in Denmark but not in Sweden. This may be the result of more intense HPV vaccination programs, in particular early catchup vaccination of young adults, in Denmark as compared to Sweden.

Recurrent respiratory papillomatosis (RRP) is a generally benign, self-limiting disease, characterized by the appearance of papillomatous lesions anywhere in the aerodigestive tract; however, the vast majority of lesions (>95%) are detected in the larynx [1, 2]. Most RRP cases (>90%) are caused by low-risk human papillomavirus (HPV) types 6 and 11 [1] and are defined as juvenile or adult onset depending on age at symptom debut. Although RRP is rare, its associated health and economic burden is substantial and symptoms include hoarseness, cough, and shortness of breath. Nearly 20% of RRP patients experience aggressive disease requiring >40 lifetime procedures, and some patients may undergo more than 100 surgeries in their lifetime [3]. Furthermore, malignant transformation of RRP occurs in 3% to 7% of patients [4].

RRP occurring in children, so-called juvenile-onset RRP (JoRRP) is most often diagnosed between ages 2 and 4 years [3] and is caused by HPV, likely transmitted from mother to child during labor as children born to mothers with anogenital warts (AGW, caused by HPV6/11) were found to have ∼230-fold increased risk of JoRRP compared to children born to mothers without AGW [5]. Studies focused on JoRRP have reported incidences ranging from 0.2 to 4.3 per 100 000 across several countries, including Canada, Denmark, Norway, South Africa, and the United States [6–13]. Adult-onset (AoRRP) incidence appears to be dominated by sexually acquired oral HPV infection transmitted horizontally between adults [14]. A limited number of studies have reported AoRRP incidence at 0.5 per 100 000 in Norway to 1.8 per 100 000 in the United States [8, 11, 12, 15].

Given the high morbidity and the potential for malignant transformation associated with RRP, it would be highly beneficial if primary prevention could be achieved. HPV vaccines have been available since around 2007 and quickly led to significant declining trends in anogenital warts caused by HPV 6/11 [16].

Thus, we hypothesized that it might indeed be possible to observe potential effects of vaccination programs on population-level of RRP incidence, given that population-wide decreases in the other major consequence of low-risk HPV (anogenital warts) were evident already in 2008 through 2010 [17, 18]. This was due to the existing female-only HPV vaccine efforts, with around a year's delay in men compared to women that could only have derived from herd protection effects as direct vaccination of men was approved, but virtually nonexistent [17].

Because of the rarity of both forms of RRP, real-world data generated from high-quality registers over disease outcomes in the Nordic countries could serve as an excellent alternative to inform studies on RRP incidence before, during, and after vaccine introduction. Indeed, potential declines in JoRRP have been observed in countries adopting quadrivalent/nonavalent vaccines [10, 11, 19–22].

In Denmark, the quadrivalent HPV vaccine (targeting HPV6/11/16/18) was licensed in 2006 and included in the Danish free-of-charge childhood vaccination program for 12-year-old girls in 2009. The first free-of-charge catch-up program started in October 2008 for girls aged 13 to 15 years. In 2012, the second free-of-charge catch-up vaccination program was initiated including women up to 27 years of age. The quadrivalent HPV-vaccine was used until January 2016. Subsequently, the bivalent HPV vaccine (targeting HPV16/18) was used for a short period until replaced by the nonavalent vaccine (HPV6/11/16/18/31/33/45/52/58) in November 2017. The Danish HPV vaccination program was extended to include 12- to 17-year-old boys in November 2019. High coverage rates have consistently been achieved at around 80% [23].

In Sweden, the quadrivalent HPV vaccine was likewise approved in late 2006 and was then offered at a subsidized price for girls aged 13 to 17 during years 2007 through 2011, which achieved a moderate coverage of around 25% [24]. A national school-based vaccination program was launched in 2012, where (after an official national tender procedure), the quadrivalent vaccine was incorporated as free of charge for all girls, typically at 11 years, and in a catchup program up until 18 years in most regions during 2012 through 2015. The national program switched to the nonavalent vaccine in 2019 and was expanded to boys in 2020. Coverage in the school-based program has consistently been higher than 80%, with the latest estimates at 90% [25].

The aim of our study was to assess time trends in the incidence of RRP in juvenile (0-14 years) and adolescent/young adult (15-29 years) forms, in Sweden and Denmark, in the pre-HPV vaccination and post-HPV vaccination period.

## METHODS

### RRP Case Definition

We performed the same data collection in parallel in 2 countries (Denmark and Sweden) in the Nordic region, where healthcare systems are similar, yet display discrete national variations in coding practices. Thus, we developed 2 nation-specific slightly adapted algorithms to best use available register data to reflect each country's own routine(s) for diagnosing RRP. All registers used are mandatory, meaning records are virtually complete for both nations since the inception of each data source.

In Denmark, the Danish Pathology Registry contains information on all cytological and histological diagnoses performed at Danish pathology departments. The registry is considered complete from 1997 and onward, but in addition, many departments have included historical data back to 1970. Using this registry, we identified all individuals with a first-time histologically confirmed occurrence of RRP, based on topography code T24 (i.e., site coding for larynx including epiglottis and vocal cords) in combination with at least 1 record of an appropriate SNOMED code for papillomas (M69790, M80500, M80520, M80530, M80600, Æ33406, Æ33411, M76700, M76701, and/or M76720) [11].

In Sweden, the Swedish In- and Outpatient Registry records all inpatient diagnoses since 1987 and all outpatient diagnoses since 2001. We opted to begin our follow-up in 2000, using inpatient data for this year and then adding outpatient data from 2001 and onward. Using this registry, we identified all Swedish individuals recorded as receiving a first diagnosis of International Classification of Diseases-10 code D14.1A/laryngeal papilloma (i.e., a combination of site topography code for larynx and morphological code for papilloma).

### Baseline Population Counts and Study Periods

The calculations of incidence rates were performed using end-year population counts for each calendar year as downloaded from publicly available national statistics agencies in the 2 countries: Statistics Denmark (www.dst.dk) and Statistics Sweden (www.scb.se). For integrity preservation purposes, RRP case numbers and incidences were presented by calendar year grouped into categories. These varied slightly per country, as the start date of registries, as well as end date available varied in each national registry at time of data collection. For Denmark, calendar years were grouped as 2000-2002, 2003-2005, 2006-2008, 2009-2011, 2012-2014, 2015-2017, 2018-2020, and 2021-2023. For Sweden, calendar years were grouped as 2000-2002, 2003-2005, 2006-2008, 2009-2011, 2012-2014, 2015-2017, and 2018-2021.

We defined JoRRP as occurring between 0 and 14 years, and opted for a lower limit of 15 years of age for AoRRP, as the median sexual debut age in Denmark and Sweden is 16 to 17 years [26]. We opted for an upper limit of 29 years for AoRRP, as males aged up to 29 years have previously shown population-level impact against the related outcome AGW, and because the uptake of HPV vaccination above age 30 years has historically been very low in both countries.

### Statistical Analysis

Incidence rates were calculated as number of RRP cases per 100 000 individuals, stratified by sex, age group (0-14 and 15-29 years) and calendar periods as defined previously.

Trends in incidence rates were summarized as average percentage changes (APCs) and 95% confidence intervals (CIs). APCs with 95% CIs were estimated using log-linear Poisson regression with a log link and log(person-years) included as an offset. Calendar year, grouped into 3-year categories because of the low number of cases preventing analyses by individual calendar year, was modeled as a continuous variable.

To allow for a change in trend around implementation of vaccination programs against HPV, a piecewise linear model with a prespecified knot in 2009 through 2011 was specified. To explore whether the coronavirus pandemic starting in 2020 may have affected trends, we also performed a sensitivity analysis ending the study period December 31, 2019. A negative APC corresponds to a decreasing trend, and a positive APC corresponds to an increasing trend. All tests were 2-sided and a *P* value of <.05 was deemed statistically significant. Statistical analyses were conducted using SAS version 9.4 (SAS Institute Inc., Cary, NC, USA).

### Ethical Approvals

Ethical approval was granted by the Swedish Ethical Review Authority (id number 2023-01631-02), which determined that informed consent from the participants was not required. As the Danish part of the study is entirely based on data from the registries, with no contact with study participants, ethical approval is not required according to Danish law. Danish data related to the project are stored at the Danish Cancer Institute's project database at Statistics Denmark (project number 704976) where all the statistical analyses were performed. In agreement with the General Data Protection Regulation, the project is registered in the Danish Cancer Society's internal list of projects dealing with personalized data (project number 2023-DCRC-0003).

### Patient Consent Statement

This study does not contain factors necessitating patient consent.

## RESULTS

### Incidence of RRP by Country, Sex, and Age of Onset

Incidence of first-time occurrence of RRP diagnoses underlying our incidence rate calculations and APC analyses are shown in Table 1. The highest incidence rates observed derived from AoRRP in men, in both Denmark (2000-2023) and Sweden (2000-2021) (Table 1).

**Table 1.** Incidence (in Case Numbers and Rate) of First-time Diagnosis of Recurrent Respiratory Papillomatosis (RRP) in Females and Males in Denmark and Sweden During the Study Period, Stratified by Sex and Age Group at Onset (0-14 Years and 15-29).

		0-14 Years (JoRRP)				15-29 Years (AoRRP)			
		Girls		Boys		Women		Men	
Country	Time period	n	IR per 100 000 py	n	IR per 100 000 py	n	IR per 100 000 py	n	IR per 100 000 py
Denmark	Baseline 2000-2011	9	0.15	12	0.19	19	0.33	52	0.88
	Postvaccine period 2012-2023	9	0.16	8	0.14	12	0.19	35	0.52
Sweden	Baseline 2000-2011	14	0.15	12	0.12	12	0.11	62	0.60
	Postvaccine period 2012-2021	17	0.20	15	0.17	36	0.40	66	0.68

Abbreviations: AoRRP, adult-onset recurrent respiratory papillomatosis; CI, confidence interval; JoRRP, juvenile-onset recurrent respiratory papillomatosis; py, person-years.

### Incidence of RRP Over the Study Period by Country, Sex, and Age of Onset

The incidence of JoRRP was very similar in both countries, both in the baseline and the postvaccine program introduction period: at 0.15 to 0.20 for girls and 0.12 to 0.19 for boys (Table 1).

The incidence of AoRRP was lower in the postvaccine introduction period in both Danish women (down from 0.33 to 0.19/100 000 person-years) and men (down from 0.88 to 0.52/100 000 person-years). By contrast, the incidence of AoRRP was higher in Swedish women in the later era (up from 0.11 to 0.40/100 000 person-years) and somewhat higher in Swedish men (up from 0.60 to 0.68/100 000 person-years) (Table 1).

### APC in RRP Over the Study Period

**Figure 1.**
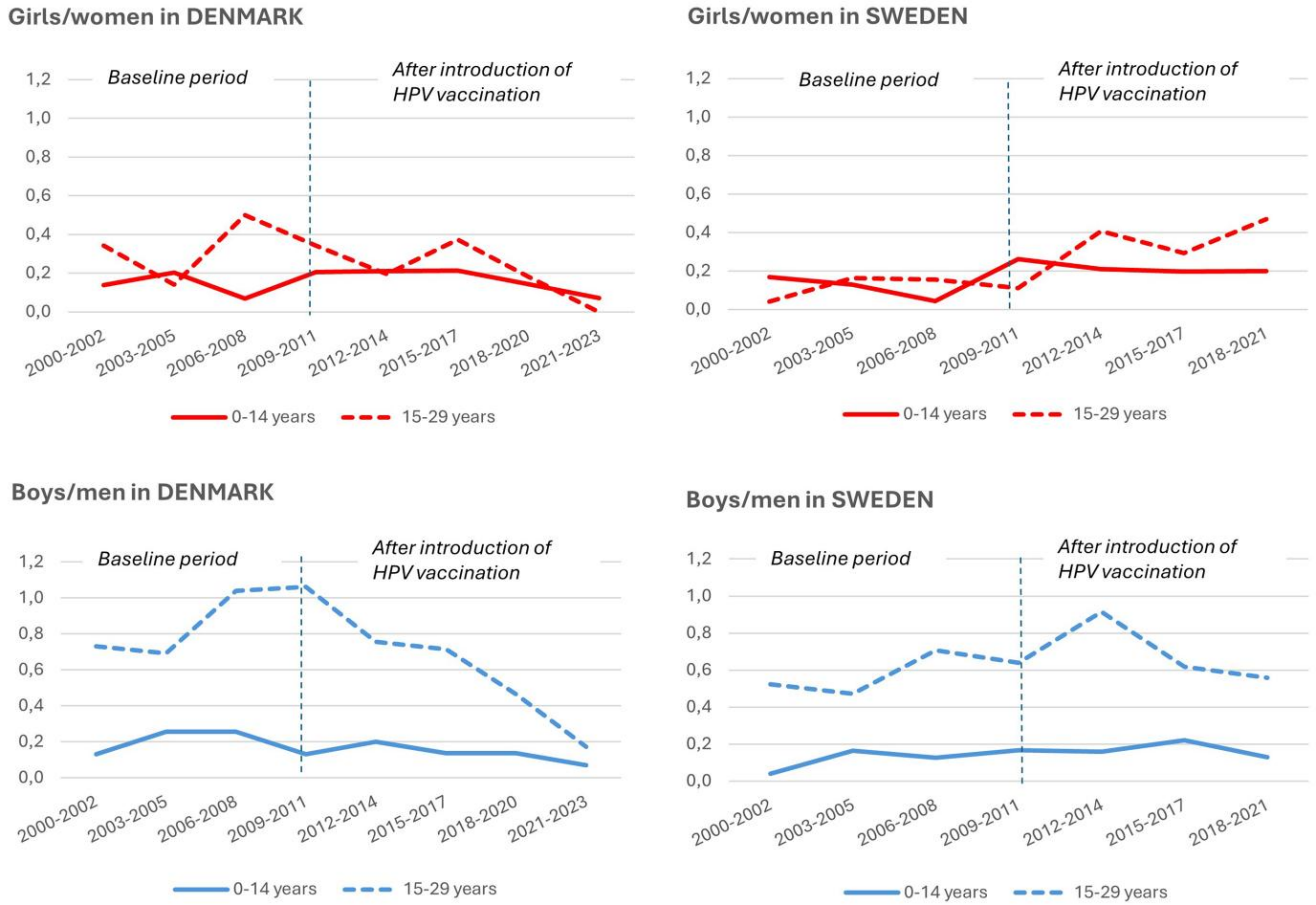
Incidence per 100 000 person-years (y axis) of first diagnosis of recurrent respiratory papillomatosis (RRP) with juvenile onset (JoRRP; 0-14 years) and adult onset (AoRRP; 15-29 years) in Denmark and Sweden after the year 2000, categorized by 3-year intervals (x axis). The dashed line indicates the calendar period 2009-2011, during which the 2 countries introduced school-based HPV vaccination programs.

The incidence of JoRRP remained rather stable during the follow-up period in both girls and boys in both countries (Figure 1). In Denmark there was a small tendency of a decreasing trend after 2009 through 2011 with estimated percentage changes per year of −4.4 (−16.2; 9.0) among girls and −6.7 (−18.5; 6.8) among boys (Table 2).

**Table 2.** Annual Percentage Changes (APC) With 95% Confidence Intervals (CI) of RRP Incidence in the Period Before Introduction of School-based HPV Vaccination Programs, and the Period After, Respectively; a Positive APC Indicates an Increasing Trend and a Negative APC Indicates a Decreasing Trend.

		0-14 Years (JoRRP)				15-29 Years (AoRRP)			
		Girls		Boys		Women		Men	
	Time period	APC (%)	95% CI	APC (%)	95% CI	APC (%)	95% CI	APC (%)	95% CI
Denmark	Baseline 2000-2011	4.7	−11.9 to 24.5	1.2	−13.2 to 18.0	4.6	−7.5 to 18.3	5.6	−1.8 to 14.1
	Postvaccine period 2012-2023	−4.4	−16.20 to 9.03	−6.7	−18.5 to 6.8	−**11.5**^a^	−**20.9 to** −**0.9**	−**11**.**0**^a^	−**16.6 to** −**4.9**
Sweden	Baseline 2000-2011	5.0	−9.00 to 21.1	10.5	−5.9 to 29.7	10.8	−4.6 to 28.6	5.4	−1.6 to 12.8
	Postvaccine period 2012-2021	0.6	−10.5 to 13.0	−2.0	−13.3 to 10.8	**9**.**8**^a^	**0.3-20.3**	−2.8	−8.3 to 3.0

Abbreviations: AoRRP, adult-onset recurrent respiratory papillomatosis; APC, annual percentage change; CI, confidence interval; JoRRP, juvenile-onset recurrent respiratory papillomatosis; py, person-years.

^a^Numbers marked in bold denotes statistically significant APC.

Regarding the incidence of AoRRP, the pattern in Denmark and Sweden differed with decreasing trends in Denmark and increasing or stable trends in Sweden in both sexes within the past decade (Figure 1 and Table 2).

In Denmark, the incidence decreased with around 11% per year in 2009 to 2023 in both women (−11.5%; 95% CI, −20.9 to −0.9) and men (−11.0%; 95% CI, −16.6 to −4.9). In contrast, female AoRRP incidence showed an increasing trend of almost 10% per year from 2009 to 2021 in Sweden (9.8%; 95% CI, 0.3-20.3; Table 2).

Our sensitivity analysis, which ended follow-up in 2019, showed results in line with those reported previously, although with lower precision. The female AoRRP incidence in Denmark still showed a decreasing trend with around −2% per year in 2009 to 2019 (−2.0%; 95% CI, −14.4 to 12.2). The female AoRRP incidence in Sweden still showed an increasing trend of 11% per year from 2009 to 2019 (11.2%; 95% CI, 1.8-21.6).

## DISCUSSION

Our ecological study is the first to show and compare the sex- and age-specific incidence of RRP over the past 20 years in 2 highly similar Nordic countries, Denmark and Sweden. We found that JoRRP (ages 0-14 years) was and remains a very rare disease, with incidence rates of 0.12 to 0.20/100 000 person-years in children of both sexes. We also found that AoRRP (ages 15-29 years) was rare in the baseline period before HPV vaccination implementation, with incidence rates of 0.11 to 0.40/100 000 person-years in women and 0.52 to 0.88/100 000 person-years in men. In both countries, the incidence of AoRRP was higher in men than women during the study period. In Denmark, in the period after vaccine program introduction, the incidence of AoRRP decreased in both men and women, whereas in Sweden findings were mixed.

With the advent of vaccines targeting low-risk HPV types, the interest in RRP has risen because of the new hope for reduced suffering among patients and families. Denmark has had a successful multicohort HPV vaccination program since 2009 with initiation of vaccination at 80% to 90% in female birth cohorts (birthyears 1985 and onward) and 70% to 80% in male cohorts (birthyears 2006 and onward). The Swedish HPV vaccination program initially targeted fewer birth cohorts and achieved lower coverage and, thus, did not use a multicohort approach. It seems likely that the decreasing incidence in Denmark could be attributed to intensive HPV vaccination and herd protection. This theory is consistent with the observation showing more rapid decline of genital warts in Denmark compared to Norway; Norway, like Sweden, has a much lower multiage cohort coverage of HPV vaccines [23]. Also, studies in the United States and Australia have shown significant declines of JoRRP after achieving successful catchup vaccination of young adult women (reviewed in [27]). Lower coverage achieved in vaccination of Swedish adolescents (only 25% in the early adopter age group of 13-17 years of age) may explain the absence of incidence reduction on the population level in Sweden.

Studies evaluating national data on HPV vaccine exposure linked on the individual level to RRP diagnosis could inform this issue and are under way to disentangle these trends.

The strengths of our work include the high quality of long-term national register data available from both countries, which are of the same structure and comprehensive nature. We used 2 slightly different country-specific algorithms to accurately identify RRP cases, because even though the registries are similar, national pathological practices are not identical. The respective algorithms were agreed with local specialists in otorhinolaryngology, which manage these patients. It should be noted that the Danish algorithm depended on histological confirmation, which meant that cases where no biopsy or surgery had been performed would not have been included. We believe these should be rare. Also, our Swedish algorithm did not include any requirement for viral testing, which meant that some HPV-negative cases may have been included. However, this should be reasonable as such RRP cases are a known occurrence in clinical practice [28]. Ultimately, we arrived at comparable estimates of incidence across the 2 countries, which is reassuring. Like others [29], we could have chosen to focus on the global code of D14.1 (benign neoplasm of the larynx). However, this would have allowed for potential misclassification because of other benign conditions (e.g., inflammation, edema, granulomas) being included in this code [11], which would have gone against our goal of accurately identifying RRP. This likely lowered the sensitivity of our combined algorithms (i.e., some cases of RRP may have been missed) but likely also increased the specificity of said algorithms (i.e., increased the likelihood that a case classified as RRP reflected a true result).

Recent publications on RRP from Sweden and Denmark indeed used similar register-based codes and definitions [11, 28], and the previous Danish study by Nielsen et al reached highly similar incidence rates as our current 2-country comparison.

Nielsen et al reported on trends in RRP reaching back to 1994, and ended in 2021, and defined AoRRP as age 18 years or older, whereas we covered the period 2000 through 2023 and opted for a definition more centered on age of sexual debut—this to reflect a potentially more adult mode of oral HPV transmission rather than a potentially higher probability of having gotten HPV from the mother. Both approaches are equally valid, but from a vaccination point of view, recommendations may want to differentiate in terms of whether to emphasize vaccination of the child or the parent. Our publication adds 2 key years of vaccine impact to the previous Danish study, and provides a Swedish counterpart, which to the best of our knowledge is the first nationwide study in the literature of this kind.

The limitations of our study include the lack of outpatient data on RRP visits during the calendar year of 2000 (the Outpatient register only starting in 2001) and the shorter follow-up in Sweden—future work should focus on this and potential updates in population-wide vaccine impact. We opted to focus on the age group below 30 years, as this is the segment of the population where we previously observed population-level declines in anogenital warts [17], but future studies should focus on RRP also in older adults and geriatric patients. Also, our study included the pandemic period of 2020 through 2022, during which elective healthcare visits were reduced. We cannot rule out the risk for some underreporting—and thus some underestimation of disease incidence—during the pandemic. However, the observed decrease we report in Denmark was apparent already from 2015-2017, which would go against the pandemic having substantially biased our results. Thus, we judge that these limitations do not appear to greatly impact the overall time perspective and lack of clear trend. Finally, we could not at this stage include individual HPV vaccination status, as our objective was to establish what the population-level incidence of RRP actually is in our respective countries. Vaccine effectiveness studies are possible but highly power-demanding and are underway separately.

Based on the present study, including 2 Nordic countries, we conclude that RRP in children and young adults appears to be nearly eliminated in Denmark, the country that adopted the most ambitious and high-coverage multiage cohort HPV vaccine strategy first. In Sweden, statistically significant population-level decreases are not yet apparent. Continued close vigilance is necessary, and we are currently pursuing studies with longer follow-up and based on individually linked HPV vaccine information, to increase our understanding of this complicated condition.
